# Structure and activity of the DHNA Coenzyme-A Thioesterase from *Staphylococcus aureus* providing insights for innovative drug development

**DOI:** 10.1038/s41598-022-08281-2

**Published:** 2022-03-12

**Authors:** Aline Melro Murad, Hévila Brognaro, Sven Falke, Jasmin Lindner, Markus Perbandt, Celestin Mudogo, Robin Schubert, Carsten Wrenger, Christian Betzel

**Affiliations:** 1 Institute of Biochemistry and Molecular Biology, Laboratory for Structural Biology of Infection and Inflammation, University Hamburg, c/o DESY, Build. 22A, Notkestraße 85, 22603 Hamburg, Germany; 2 Unit for Drug Discovery, Department of Parasitology, Institute of Biomedical Sciences, University of São Paulo, Avenida Professor Lineu Prestes 1374, São Paulo, SP 05508-000 Brazil; 3 The Hamburg Centre for Ultrafast Imaging, CUI, Luruper Chaussee 149, 22761 Hamburg, Germany

**Keywords:** Drug discovery, Structural biology

## Abstract

Humanity is facing an increasing health threat caused by a variety of multidrug resistant bacteria. Within this scenario, *Staphylococcus aureus*, in particular methicillin resistant *S. aureus* (MRSA), is responsible for a number of hospital-acquired bacterial infections. The emergence of microbial antibiotic resistance urgently requires the identification of new and innovative strategies to treat antibiotic resistant microorganisms. In this context, structure and function analysis of potential drug targets in metabolic pathways vital for bacteria endurance, such as the vitamin K_2_ synthesis pathway, becomes interesting. We have solved and refined the crystal structure of the *S. aureus* DHNA thioesterase (*Sa*DHNA), a key enzyme in the vitamin K_2_ pathway. The crystallographic structure in combination with small angle X-ray solution scattering data revealed a functional tetramer of *Sa*DHNA. Complementary activity assays of *Sa*DHNA indicated a preference for hydrolysing long acyl chains. Site-directed mutagenesis of *Sa*DHNA confirmed the functional importance of Asp16 and Glu31 for thioesterase activity and substrate binding at the putative active site, respectively. Docking studies were performed and rational designed peptides were synthesized and tested for *Sa*DHNA inhibition activity. The high-resolution structure of *Sa*DHNA and complementary information about substrate binding will support future drug discovery and design investigations to inhibit the vitamin K_2_ synthesis pathway.

## Introduction

The increase in hospital-acquired infections (HAI) is one of the major concerns for the global health system. Bacterial infections acquired during patient hospitalization contribute not only to significant mortality, but also usually require additional therapies, increasing even more the financial burden for the healthcare systems^1^. Among bacterial infections involved in HAI, *Staphylococcus aureus* is a leading pathogen found in hospitals causing serious bacteraemia leading to sepsis and to infection of internal organs, such as the heart, lungs and joints, usually occurring after invasive procedures like introducing implantable medical devices^2^.

The first successful treatment of *S. aureus* infections, in the 1940’s, involved mainly the administration of β-lactam antibiotics like penicillin G. Targeting the bacterial enzymes of the cell wall biosynthesis, called penicillin-binding proteins (PBPs), the inhibition of the PBPs induced by β-lactam antibiotics interferes with the cross-linking of peptidoglycan, making the cell wall mechanically fragile and the cell to perish^3^. However, since penicillin resistance was first discovered in the early 1950s due to the production of specific β-lactamases by the acquisition of the *blaZ* gene that is capable of hydrolyzing β-lactam antibiotics, the phenomenon of drug resistance has been observed for an increasing number of Gram-negative and Gram-positive pathogens^4^.

In the early 1960s, the structure of natural penicillin has been modified allowing the development of a new antibiotic named methicillin, a semi-synthetic penicillinase-resistant β-lactam antimicrobial to replace the conventional penicillin treatment. Methicillin was noted due to inactivation resistance by penicillinases and activity against several penicillinase-producing staphylococci. However, the widespread use of methicillin has led to the emergence of Methicillin resistant *S. aureus* (MRSA) by acquisition of a non-active gene encoding a PBP2a which has a lower affinity for β-lactam antibiotics^5^. Nowadays, *S. aureus* exhibits resistance not only to methicillin but also to a vast number of β-lactam antimicrobial agents, including carbapenems and cephalosporins, until the most recent antibiotic such as linezolid^6^. The increase threatening of multidrug resistant *S. aureus*, in particular MRSA, unfortunately has narrowed therapy options substantially, making the treatment of MRSA infections even more complex.

Further, *S. aureus* has a particular ability to respond quickly to new antibiotic treatments. During antimicrobial treatment, the selective pressure caused by antibiotics can induce the generation of subpopulations of *S aureus* with slower growth compared to the wild variant, called small variant colonies (SCVs)^7^. The presence of SCVs is often associated with persistent and recurrent infections, which are difficult to diagnose and treat with antimicrobials. Studies have shown that SCVs are associated with disturbances in the electron transport chain caused by a class of antibiotics that acts on polypeptide synthesis. Deficiency in the respiratory chain can reduce the influx of antibiotics by the bacteria and, consequently their susceptibility to antimicrobial treatment^8,9^. The most frequent SCV phenotypes are mutations in genes involved in the biosynthesis of components required for the electron transport chain, reducing the emergence of new therapies for MRSA.

In order to overcome bacterial resistance identification of novel drug targets as well as the development of innovative antibiotics with high specificity and effectiveness is needed. The latest drug discovery investigations are focusing on obtaining structural knowledge about enzymes involved in the bacterial cell metabolism which are critical and vital for bacteria endurance. The lipid-soluble compound vitamin K_2_ (menaquinone), with about 85–90% almost entirely located in the bacterial membrane, is an important electron carrying component in the membrane-bound complexes of the electron-transport chain (ETC) pathway in cell respiration^10^. Since the cellular respiration in humans does not involve the use of menaquinone and the ETC pathway in bacteria is an indispensable and essential component for ATP production, bacterial electron-transport enzymes have shown a substantial potential for novel drug development investigations. Humans entirely depend on the uptake of vitamin K which is known to improve health in cardiovascular disease, chronic kidney disease and bone metabolism.

In bacteria, several enzymes involved in menaquinone de novo biosynthesis have already been analysed, e.g. *Escherichia coli* MenD^11^, *Mycobacterium turbeculosis* MenB^12^ and *Pseudomonas* sp. 4-Hydroxybenzoyl- CoA thioesterase^13^. In a recent study by Smith et al*.* (2021) the MenI (DNHA Thioesterase) gene was deleted in *Listeria monocytogenes* and data obtained showed clearly the vital contribution of DHNA-CoA for the bacterial replication in vitro, ex vivo, and in vivo, confirming the specific role of DHNA in promoting bacterial survival in the cytosol of macrophages and demonstrating the need of the DHNA for menaquinone biosynthesis, cytosolic survival, and virulence^14^.

Based on structural information of *E. coli* MenE, Matarlo et al*.* (2015) designed several *o*-succinylbenzoyl (OSB) secondary amine analogues (OSB-AMS) with high specificity and showing antimicrobial activity^15^. Furthermore, the effect of these OSB-AMS on vitamin K levels of *S. aureus* can be assigned to direct interference of menaquinone biosynthesis. According to the authors, the identification of a putative interaction of OBS-AMS and the arginine Arg222 of *Sa*MenE, and corresponding mutagenesis studies confirmed the importance of Arg222 in substrate binding as well as the mechanisms of inhibition of *Sa*MenE^15^. Furthermore, Choi and collaborators, also designed, synthesized and evaluated several inhibitors based on the MenA structure and revealed promising inhibitory activity with low minimum inhibitory concentrations (MICs) ranging from 1 to 8 μg mL^−1^ against MRSA strains^16^. These observations highlight the significance of the menaquinone pathway for *S. aureus* and demonstrate that structural investigations of involved ETC key enzymes may lead to interesting data supporting drug discovery.

In this context we solved and refined the tetrameric structure of 1,4-dihydroxy-2-naphthoyl coenzyme A thioesterase—*Sa*DHNA (E.C. 3.1.2.28) from *S. aureus* to 1.3 Å resolution, which in combination with site-directed mutagenesis allowed the identification of essential residues for thioesterase activity and substrate binding.

## Results

### Three-dimensional structure of the tetrameric *Sa*DHNA

Crystals of the native protein belong to the space group *P*2_1_ with unit cell dimensions of a = 53.61, b = 90.66, c = 75.38 Å, α = γ = 90 and β = 92 (°) with four molecules in the asymmetric unit. The calculated Matthews coefficient is 2.4 Å^3^ Da^−1^, corresponding to an approximate solvent content of 48%. The *Sa*DHNA monomer comprises 155 amino acids with a corresponding molecular weight of 18.1 kDa and a theoretical pI of 5.5. The structure of the *Sa*DHNA tetramer was refined to *R*_work_/*R*_free_ (%) values of 17.8/19.8, respectively. Data collection statistics and final refinement parameters are summarized in Table 1. Residues 156–165, corresponding to the strep-tag II at the C-terminus (SA-WSHPQFEK), as well as the residues DGIDSL at the C-terminus of chain B and D were disordered and not included in the final model. The atom coordinates of *Sa*DHNA were deposited in the Protein Data Bank with pdb code 6FDG.

**Table 1:** Data collection, processing and refinement statistics.

	Native data	Deriv. data
**Data collection**^**a**^		
Beamline	P13, PETRA III (DESY, Germany)	P14, PETRA III (DESY, Germany)
Wavelength (Å)	1.0332	1.072
Space group	*P*2_1_	*P*2_1_
**Unit-cell parameters**		
*a* (Å)	53.61	55.20
*b* (Å)	90.78	90.90
*c* (Å)	75.38	74.80
*α* = *γ* (°)	90	90
*β* (°)	92.0	90.8
**Resolution range (Å)**	75.33–1.3 (1.33–1.3)	55.29–2.00 (2.1–2.0)
Completeness (%)	97.9 (96.1)	98.2 (94.5)
*R*_merge_ (%)	4.0 (123)	11.5 (89.8)
Multiplicity	6.9 (7.0)	6.9 (6.6)
〈 *I*/σ(*I*)〉	19.9 (2.0)	12.7 (2.7)
**Refinement**		
Resolution range (Å)	75.3–1.3	
No of reflections used for refinement	164,237	
*R*work/*R*free (%)	17.8/19.8	
**No. of atoms**		
Protein	5483	
Water	314	
Average *B* value (Å^2^)	23.6	
**R.m.s.d**		
Bonds (Å)	0.03	
Angles (°)	2.86	
**Ramachandran plot**		
Favoured regions (%)	98.4	
Allowed regions (%)	1.6	
Disallowed region (%)	0	

^a^Numbers in parenthesis refer to the outer resolution shell.

**Figure 1:**
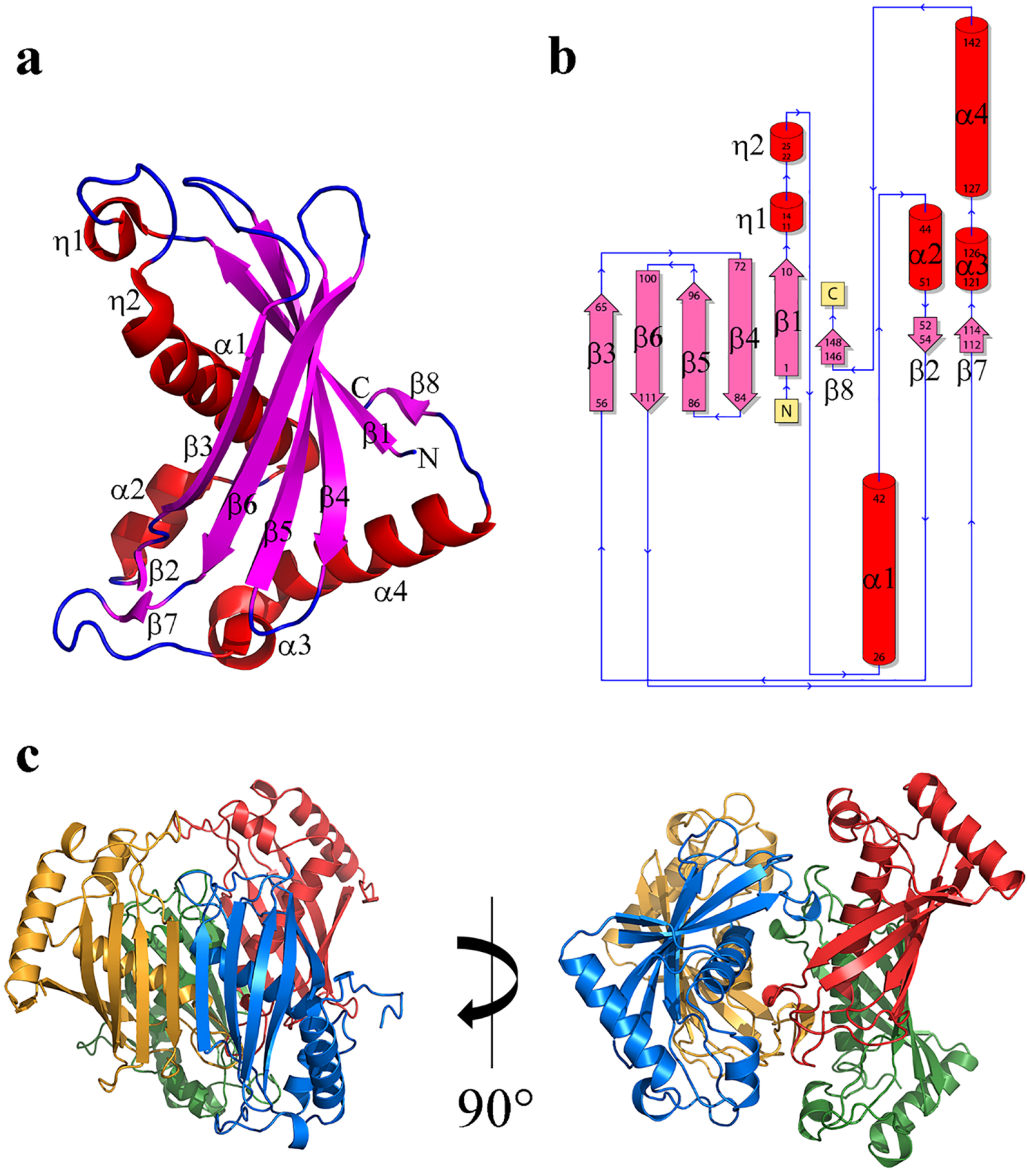
(**a**) Cartoon representation of the *Sa*DHNA monomer. (**b**) The secondary structure of *Sa*DHNA is shown schematically and annotated including the topology plot of its HotDog domain. (**c**) The quaternary arrangement of *Sa*DHNA is shown for the individual monomer chains with chain A in blue, B in yellow, C in red and D in green. The figure was created applying the program PyMOL (Molecular Graphics System, Version 1.0.5.4 Schrödinger, LLC).

The overall structure of the *Sa*DHNA monomer and its topology are illustrated in Fig. 1a,b, showing that *Sa*DHNA has a high content of compact secondary structure elements (26% α-helix and 32% β-strand) with a relatively extended five-stranded antiparallel β-sheet element and one parallel β-sheet segment in the sequential order 8-1-4-5-6-3, involving the residues Val146-Ile148 (β8), Met1-Ala10 (β1), Glu72-Ser84 (β4), Arg87-Asn96 (β5), Glu100-Ile110 (β6), Pro56-Lys65 (β3) and two short antiparallel β-sheets containing the amino acid residues Gly52-Ile54 (β2) and Ile112-Glu. The β-sheets β5 and β6 are interrupted by four β-bulges at Val89, Ile94, Asn96 and Glu108. All β-sheets wrap around the four α-helices formed by residues Tyr26-Gly42 (α1), Ser44-Gln51 (α2), Arg121-Phe126 (α3) and Pro127-Glu142 (α4) and two short α-helices comprising the residues Arg11-Glu14 (Ƞ1) and Try22-Asn25 (Ƞ2) connected by a β-turn. The turns connecting the β-strands are type I (Ser84-Arg87, Asn96-Gly99) and type IV (Phe69-Glu72, Lys113-Trr116). In addition, two β-hairpins between β-4/β-5 (class 2:4) and β-5/β-6 (class 3:5) and a γ-turn Ile152-Ser154 after the last β8 strand are observed within the structure.

Two monomers of *Sa*DHNA assemble to form a homodimer by a network of nine hydrogen bonds between strand β3 of chains A or C with the adjacent β3 strand of chains B or D, respectively, producing an overall 10-stranded antiparallel β-sheet motif involving residues Thr58-Tyr64, Asp59-Asn63, Leu60-Val62, Asn61-Asn61, Tyr64-Thr58, Asn63-Asp59 and Val62-Leu60. Three further H-bonds stabilize α2, α1 and Ƞ2, involving the residue pairs Glu49-Lys17, Glu31-Tyr22 and Tyr22-Glu31 respectively, along with two ionic interactions involving Lys17-Glu49. There are two hydrogen bonds between chains A and C stabilizing the loop involving Thr15 and Lys17. Between chains A and D four hydrogen bonds involving residues Arg11-Glu14, Glu14-Arg11, Tyr12-Glu49 stabilize interactions of Ƞ1-Ƞ2 and Ƞ1-α2 and Glu31-Ala13 stabilize α1-Ƞ1 interaction. Two ionic interactions stabilize Ƞ1 between residues Arg11-Glu14. Additionally, the main α-helix (α1) is stabilized by a number of hydrophobic interactions. All *Sa*DHNA chain interface features are summarized in Table 2. Thereby the quaternary structure of *Sa*DHNA is formed by the assembly of four identical subunits A, B, C and D, arranged as dimer of dimers, forming a homo-tetrameric structure, as shown in Fig. 1c.

**Table 2:** Summary of *Sa*DHNA chain interface features.

Chains	No. of residues located in interface regions	Interface area (Å^2^)	No. of hydrogen bonds	No. of non-bonded contacts	No. of ionic bonds
A-B	23:20	1051:1070	12	115	2
C-D	22:20	1053:1058	12	121	1
A-C	7:8	432:432	2	50	-
B-D	7:7	428:426	4	52	-
A-D	9:9	611:621	4	35	2
B-C	10:10	614:607	4	38	2

In the crystal structure four molecules of *Sa*DHNA are present in the asymmetric unit. The oligomeric state analysis performed using PDBe PISA^17^ provided a value for the buried area of each monomer at the A-B (or C-D) interface of approx. 1040 Å^2^, corresponding to 13% of the overall surface area of each monomer. The program PBEQ solver^18^ was used to calculate the monomer/tetramer electrostatic surface potential to be ΔG_elec_ of − 9516.320 kcal mol^−1^ for the tetramer and − 2676.08 kcal mol^−1^ for the monomer. In addition, the solvation energy was calculated to be ΔΔ_elec_ of 6.840 kcal mol^−1^ by utilizing the Poisson–Boltzmann Equation^19^, indicating a significant increase in stability upon tetramer formation.

**Figure 2:**
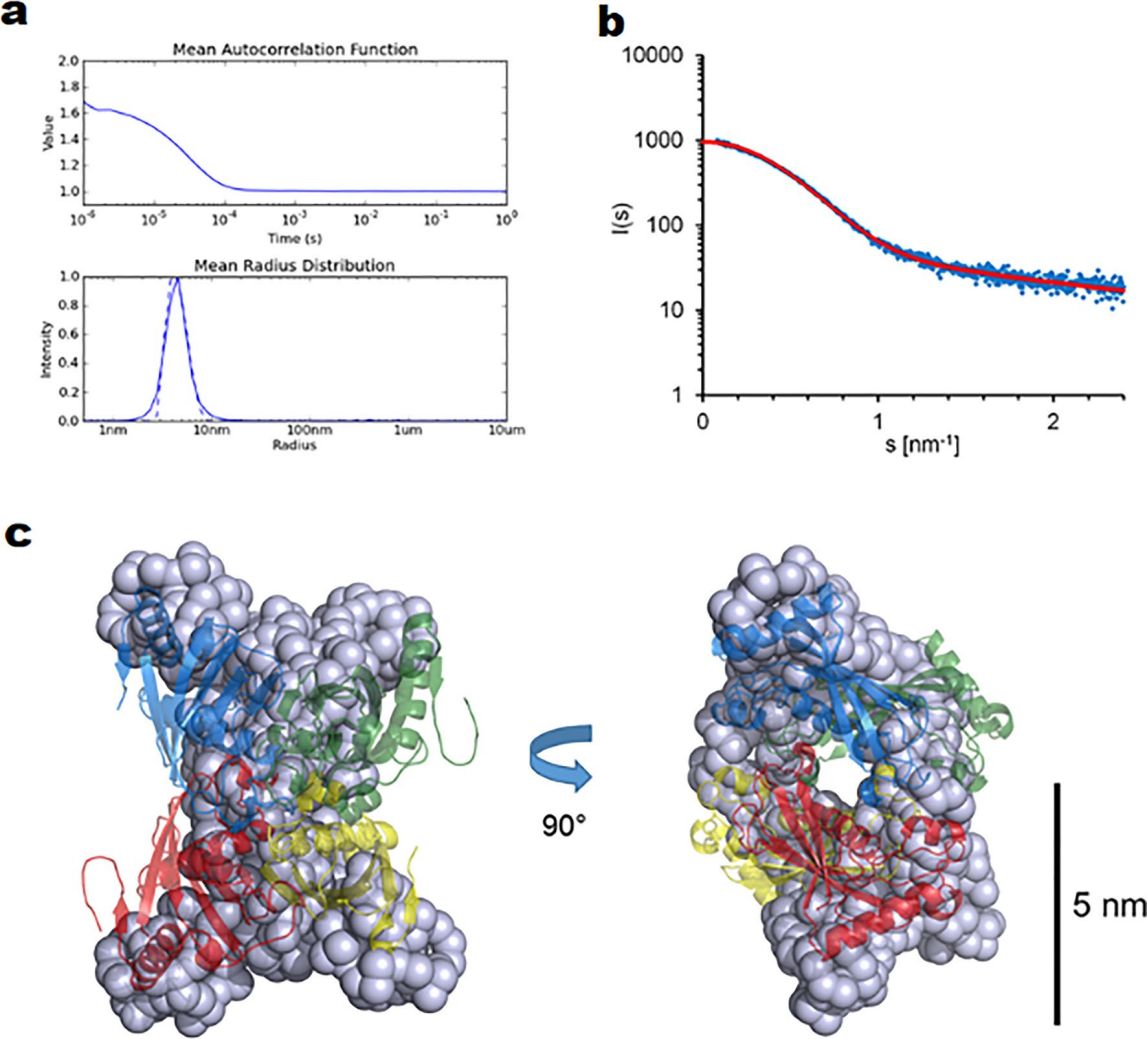
DLS and SAXS data of *Sa*DHNA. (**a**) Autocorrelation function and corresponding mean radius distribution obtained by DLS, averaged over 300 s. (**b**) Averaged X-ray scattering intensities of *Sa*DHNA (blue dots) in arbitrary units plotted against the scattering angle, i.e. momentum transfer values ranging from s = 0.05 to 2.4 nm^−1^. The red fit function corresponds to the *Sa*DHNA ab initio model displayed in figure panel C, sharing a χ^2^-value of 1.54 with the corresponding experimental scattering data. (**c**) Superimposition of a single *Sa*DHNA ab initio model calculated applying the program GASBOR and the tetramer of *Sa*DHNA revealed by the crystal structure.

In order to verify the oligomeric state of *Sa*DHNA in solution, we applied dynamic light scattering (DLS) and SAXS. DLS measurements showed a monodisperse hydrodynamic radius of 4.3 ± 0.3 nm (Fig. 2a,b) and SAXS data provided, based on the p(r) function and using the Guinier approximation, a corresponding maximum diameter for *Sa*DHNA of 10.9 nm, and the radius of gyration was calculated to be 3.27 ± 0.09 nm. Those values along with the calculated SAXS ab initio model (Fig. 2c) confirm a functional tetramer in solution with a slightly elongated twisted “butterfly-like” shape, as observed also for the crystal structure.

### Comparison with homologues enzymes

**Figure 3:**
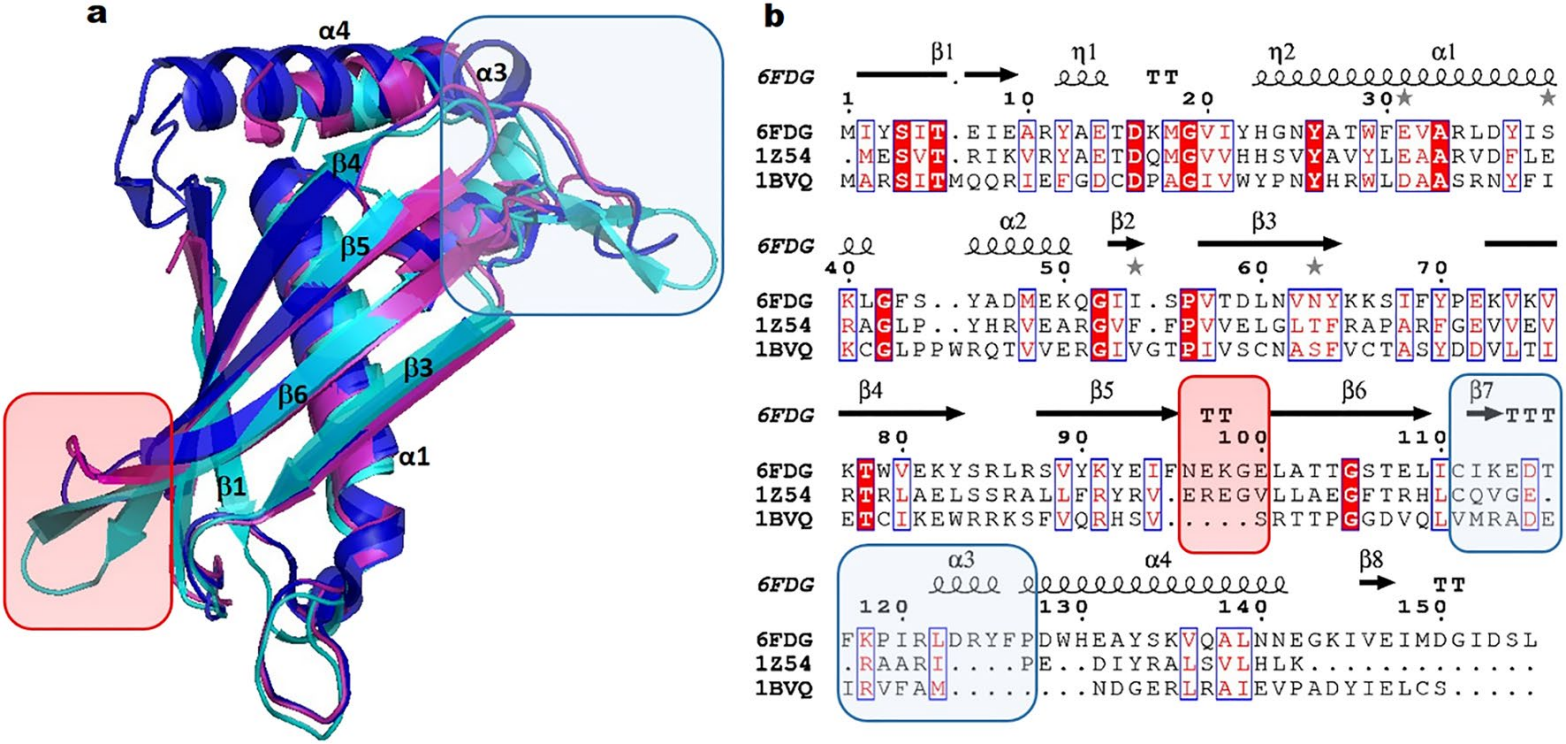
(**a**) Superimposition of *Sa*DHNA (dark blue) with homologous structures, PDB codes 1Z54 of *Thermus. thermophylus* (pink) and 1BVQ of *Pseudomonas sp* (cyan). (**b**) Protein sequence alignment. The light blue and red boxes illustrate the highest variability compared between the homologue enzymes. Multiple sequence alignment was performed applying the program ClustalOmega^20^ with default parameters.

A search to identify and compare homologous structures was performed applying the European Bioinformatics Institute database server http://www.ebi.ac.uk/msd-srv/ssm. The top-scoring domain superimposition indicated homology to a hypothetical thioesterase of *Thermus thermophilus* (pdb code 1Z54) with a Cα core r.m.s.d. of 1.1 Å for 41 aligned residues and 32% amino-acid sequence identity, along with another well characterized 4-hydroxybezoyl-CoA thioesterase from *Pseudomonas* sp. strain CBS3 (pdb code 1BVQ)^13^ with a corresponding Cα r.m.s.d of 1.7 Å and 21% sequence identity. These results confirm that *Sa*DHNA belongs to the protein family displaying a HotDog domain fold class I. The structure is most conserved in β1and in strands β3 up to β6, in helix α1 and in the short α2 helix (Fig. 3a). The highest variability was observed in the loop region connecting β5 and β6 (Fig. 3a, light red box), as well as in the region connecting β6 and α4 (Fig. 3a, light blue box). These structural differences can be assigned to the absence of two short antiparallel β sheets in the *Sa*DHNA structure, as well as the presence of the extra α-helix α3 and the elongated helix α4 including the residues Gln137-Lys144, the parallel strand β8 (Val46-Ile148) and a flexible loop (Met149-Leu155) at the C terminal region of *Sa*DHNA. The protein sequence alignment of *Sa*DHNA, shown in Fig. 3b, also revealed that Asp16 is likely to be an essential residue for a thioesterase activity (numbering according to *Sa*DHNA). This residue is conserved among the compared proteins.

### Putative active site of *Sa*DHNA thioesterase

**Figure 4:**
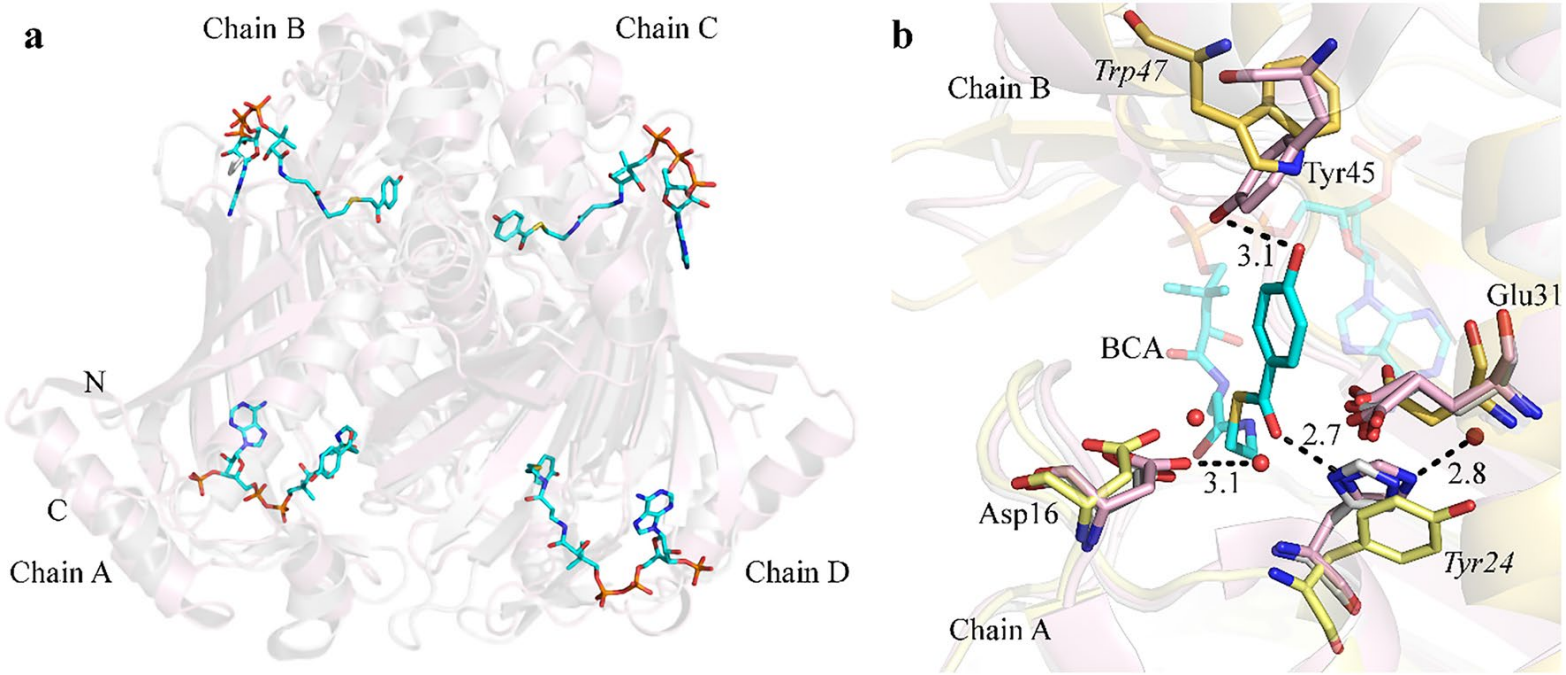
Putative active sites of the tetrameric *Sa*DHNA-CoA thioesterase. (**a**) Ribbon representation of *Sa*DHNA (light pink) superimposed with the 4-hydroxybenzoyl-CoA thioesterase D17N mutant structure of *Pseudomonas* (PDB code 1LO9) (light grey). The substrate 4-hydroxybenzoyl-CoA (BCA) complexed with the D17N *Ps*4HBT mutant is shown in stick representation; carbon in cyan; sulfur in yellow; phosphate in orange, nitrogen in blue, oxygen in red; “N” and “C” indicate N- and C-terminus, respectively. (**b**) Residues at the proposed active site region with assigned thioesterase activity of *Sa*DHNA (pink), *Pseudomonas* 4HBT structure (yellow) and hypothetical thioesterase from *Thermus. thermophilus* (pdb code 1Z54) (light gray). Italic amino acid residue labels indicate corresponding residues in the *Pseudomonas* 4HBT structure (yellow).

For future drug discovery experiments targeting *Sa*DHNA, detailed information about the location of putative active residues within the active site region and their interactions are an essential requirement. In order to obtain insights *Sa*DHNA was superimposed on the D17N mutated 4-hydroxybenzoyl-CoA thioesterase from *Pseudomonas* sp *strain CBS-*3^21^ (pdb code 1LO9) in complex with the substrate 4-hydroxybenzoyl-CoA (BCA). It can be seen that a putative active site of *Sa*DHNA is arranged by the interface region between two neighbouring monomers in the quaternary structure of a homo-tetramer, resulting in the formation of four active sites, as shown in Fig. 4a.

According to active site analysis investigations performed by Thoden et al*.*^22^, the binding of the ligand is mediated by interactions with the amino acid residues Tyr45 from one chain (B or D) and Asp16 and His23 from the corresponding neighbouring chain (A or C), all located in the interface region, as shown in Fig. 4b. According to the authors the binding of the substrate 4-hydroxybenzoyl CoA inside the binding pocket is supposed to be mainly stabilized by hydrogen bonds formed between the hydroxyl group from the aromatic moiety of the ligand and the benzoyl ring hydroxyl group of the amino acid residue Tyr45, as well as through the carbonyl carbon group from the amino acid Glu31 mediated by a water molecule. Also, the coenzyme A ribose of BCA molecule is positioned in a cavity located at the surface of one monomer and the remaining ligand is located in a deep cleft formed by the subunit–subunit interface. The position of the benzoyl ring hydroxyl group of the substrate may interact with the side chain hydroxyl group of Tyr45 from one monomer and with the side-chain of His23 located at helix Ƞ2 from the corresponding subunit. Thereby the thioester carbonyl group of the substrate is located close to the N-terminal region of α1, forming a hydrogen bond with the His23 side chain imidazole ring.

### Thioesterase activity assays and peptide binding analysis by fluorescence spectroscopy

To obtain some initial information about *Sa*DHNA thioesterase substrate specificity, two different substrates classified according to their CoA chain length and unsaturation degree were assessed. The longest acyl-CoA chain substrate contained eighteen acyl saturated chain stearoyl-CoA (C18:0), while the shortest chain contained four acyl chain crotonyl-CoA with one unsaturation (C4:1). Thioesterase activity was observed by the increase in free thiol-CoA thioester hydrolysis formation of the 2-nitro-5-thiobenzoate anion (TNB^2−^), resulting from the reaction of the thiolate anion (RS^−^) with Ellman’s reagent (DTNB^2−^) and one mixed disulphide (R-S-TNB^−^). *Sa*DHNA showed a 474-fold higher specific activity toward the extended acyl CoA chain in comparison to the short chain crotonyl-CoA (Supplemental Table S2). Furthermore, the significance of amino acid residues Asp16 and Glu31 for thioesterase activity of *Sa*DHNA was investigated by applying the longer acyl CoA chain as the substrate. The hydrolysis rate of stearoyl-CoA by the D16A mutant, which had the putative site carboxylate group removed, decreased 300-fold, while no activity was detected above the background level of stearoyl-CoA for the E31N mutant, indicating that both amino acid residues are essential for the activity of *Sa*DHNA.

In parallel, in terms of structure-based computational design investigations, the atomic structure of *Sa*DHNA was used for docking analysis applying the Bioluminate module from the Schrödinger suite, (Schrödinger, LLC, New York, 2021). Based on the structure of *Sa*DHNA, and in particular considering the putative active site (Asp16, His23, Glu31 and Try45), we designed several peptides (ranging from 5 to 6 residues) which potentially bind to *Sa*DHNA and inhibit its activity. Docking investigations identified two binding sites, one at the *Sa*DHNA surface and one in the putative active site. The most effective peptide inhibitors, named Pep-1 (YGSDGR) and Pep-2 (EGEYE), showed the smallest Optimized Potentials for Liquid Simulations (OPLS) force field (potential energy OPLS2005 − 1583.93 kcal mol^−1^ and − 1927.27 93 kcal mol^−1^, respectively)^23^.

Pep-1 (YGSDGR), with a molecular weight of 654.28 Da, was predicted to bind inside the active site region with a ΔG_bind_ of − 81.0 kcal mol^−1^. According to the docking analysis, the protein-peptide complex is mediated mainly through six hydrogen bonds formed between Pep-1 and residues present in the putative active site. The benzoyl ring of the tyrosine of Pep-1 has non-covalent π-stacking interactions with the Try45 benzoyl ring of the *Sa*DHNA structure, as well as with the side chain of Ser55 through a hydrogen bond. The residues important for the substrate binding and activity, Glu31 and Asp16, respectively, are predicted to interact with the amide of the peptide backbone and with the side chain of serine of the peptide via hydrogen bonds as well. The second peptide (Pep-2) with a molecular weight of 623.23 Da, was predicted not to interact with the residues in the putative active site region, but with residues located on the surface of the *Sa*DHNA structure, close to the binding site entrance, with a ΔG_bind_ of − 41.3 kcal∙mol^−1^. Pep-2—*Sa*DHNA interactions involve seven hydrogen bonds, as well as hydrophobic interactions between residues localized between connecting loops β2-β3, β4-β5 and β5-α3. In both cases, the predicted peptide binding free energies (− 81.0 kcal mol^−1^ and ΔG_bind_ of − 41.3 kcal mol^−1^ respectively) (Fig. S2) indicate stable protein-peptide complexes^24^. In vitro inhibition assays were performed applying 100 µM of each peptide and 10 µM of *Sa*DHNA and resulted in no detectable activity in comparison to control assay without peptides, confirming that the predicted peptides indeed inhibit the *Sa*DHNA thioesterase enzyme.

**Figure 5:**
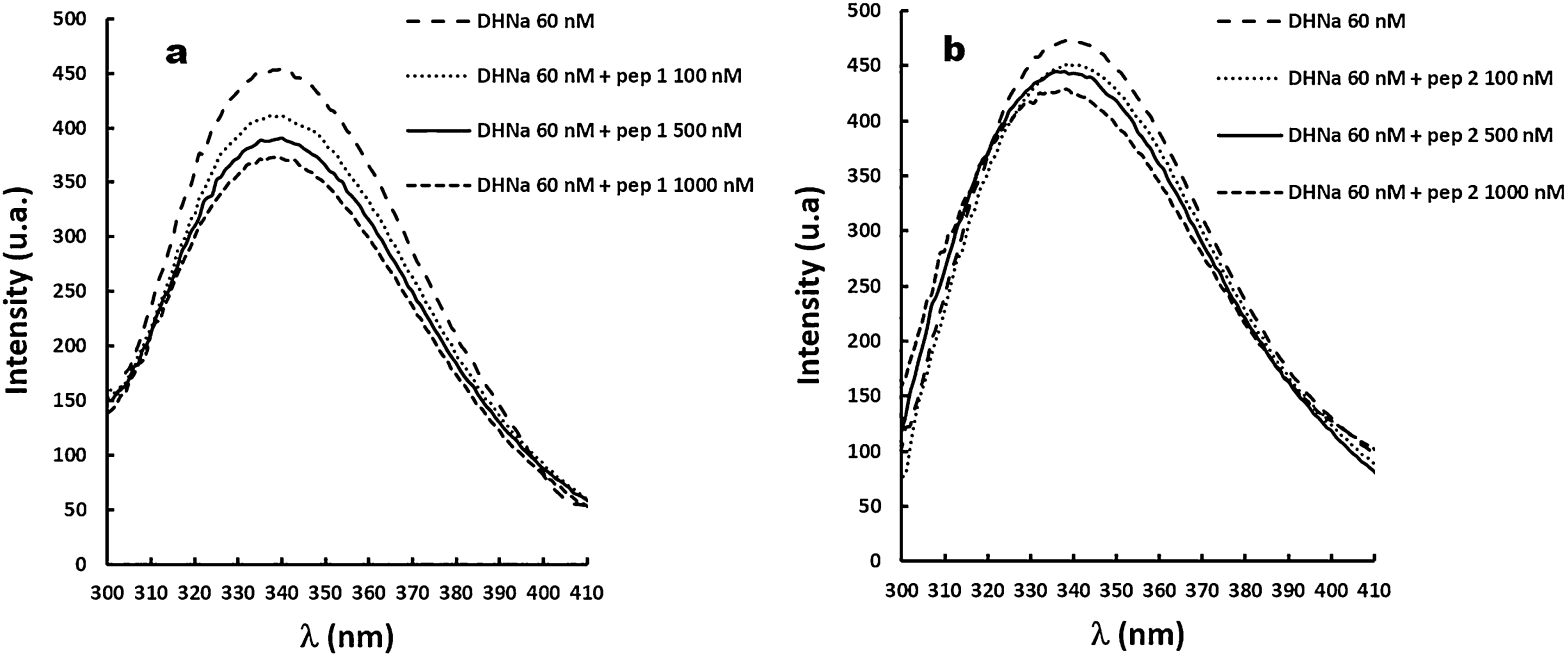
Fluorescence emission spectra of *Sa*DHNA (60 nM) in the absence and the presence of the gradual concentrations of (**a**) Pep-1 and (**b**) Pep-2 up to 1000 nM.

Fluorescence spectroscopy was employed to analyse the inhibition of *Sa*DHNA by the aforementioned peptides. The intrinsic fluorescence quenching was assessed for a gradient concentration of Pep-1 and Pep-2. The emission spectra of *Sa*DHNA in buffer only and *Sa*DHNA quenched with different concentrations of Pep-1 and Pep-2 are shown in Fig. 5a,b, respectively. The amino acid sequence of *Sa*DHNA has three tryptophan residues, W29, W79 and W129 and a corresponding maximum emission near 340 nm. The gradual increase of Pep-1 to up to 1000 nM did not shift the emission spectra maximum of *Sa*DHNA, while Pep-2 resulted in a slight blue shift of the emission, probably explained by the slight change in the polarity of the solvent surrounding the tryptophan residues^25^. A decrease in the fluorescence intensity was observed for both peptides. According to the quenching theory this may indicate a decrease in the lifetime of the excited state, corresponding to an additional rate process that depopulates the excited state and/or the formation of a non-radioactive ground state between the fluorophore and the quencher resulting in a non-fluorescence emission^25,26^.

**Figure 6:**
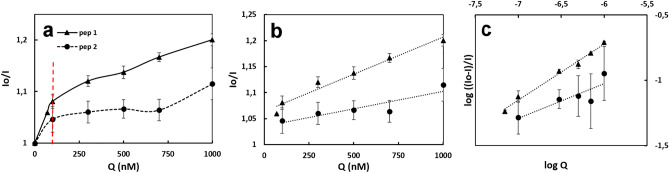
(**a**) Stern–Volmer plot for Pep-1 and Pep-2 ranging from 0 to 1000 nM. (**b**) Stern–Volmer plot indicating the bimolecular quenching constant (K_q_). (**c**) Static quenching plot to estimate the affinity-binding constant (K_a_).

The Stern–Volmer theory explains the relationship between fluorescence intensity and the presence of a quencher. The data obtained with different concentrations of peptides were analysed independently of the quenching process. Thereby the bimolecular quenching constant (K_q_) assigned to the efficiency of quenching and the binding or affinity constant (K_a_) for the associated complex were calculated^27,28^. *Sa*DHNA showed a biphasic quenching behaviour, since the Stern–Volmer plot presented two linear quenching steps delimited by the red dotted line in Fig. 6a. The first one and faster quenching step was observed up to 100 nM (left side of the red dotted line), while a second and slower quenching was observed at higher concentrations of both peptides (Fig. 6a). To investigate the quenching process and to estimate K_q_ and K_a_ the data from the second and slower step were considered (Fig. 6b,c, respectively). To estimate the bimolecular quenching constant (K_q_), the Stern–Volmer quenching constant (K_sv_) was determined from the slopes of Io/I versus Q (quencher concentration) (Fig. 6b) and multiplied by 10^–8^, which corresponds approximately to the lifetime of a biomolecule fluorophore in the absence of a quenching agent^28^. Therefore, K_q_ [M^−1^ s^−1^] for Pep-1 and Pep-2 were 13.3 × 10^12^ ± 0.6 × 10^12^ and 9.3 × 10^12^ ± 1.8 × 10^12^, respectively. According to the diffusion controlled quenching theory K_q_ values close to 1 × 10 ^10^ M^−1^ s^−1^ are associated with a process involving dynamic quenchers, whereas values of K_q_ larger than the diffusive limit indicate binding interactions between the fluorophore and the quencher^26^. Our results demonstrated K_q_ values 100-fold higher than the diffusive upper limit. Therefore, it is most likely that the quenching process obeys the static mechanism through the complex association between *Sa*DHNA and the peptides.

As the estimated K_q_ presented higher values than expected for dynamic quenching, we analysed the data applying static quenching theory to estimate the affinity constant K_a_ for the associated complex. The intercept of log ((Io-I)/I) versus log Q plot (Fig. 6c) is equal to the logarithm of K_a.._ Thereby the intercept antilog calculation determines the K_a_ values for both peptides^26^. The respective affinity constants for Pep 1 and Pep 2 were 72.7 ± 2.9 M^−1^ and 3.4 ± 0.9 M^−1^, indicating that Pep-1 has a 21-fold higher binding affinity to *Sa*DHNA than Pep-2. These results strongly indicate Pep-1 as a suitable candidate to be considered as a lead compound for further drug development investigations, to identify compounds that can directly interact with and inhibit *Sa*DHNA, an essential enzyme of the menaquinone pathway (Fig. S1).

## Discussion

We solved and refined the crystal structure of *Sa*DHNA, and assigned *Sa*DHNA to be a member of the HotDog fold class I superfamily of proteins, where all β-strands point outwards and the long main α-helix points towards to the core of the structure. *Sa*DHNA associates as a dimer of homodimers, with a face-to-face (or helix-to-helix) conformation. Leesong and co-workers firstly described the “HotDog” fold for a thiol ester dehydratase-isomerase from *E. coli,* named FabA (pdb code 1MKA)^29^. Since then, a number of proteins possessing the “HotDog” fold were described for several organisms^30–34^. Despite the absence of a consensus sequence and a sequence identity ranging only between 10 and 20%, the overall fold and N- or C-terminal secondary structure elements are similar and are particularly characteristic for members of the “HotDog” fold protein family. Although overall low sequence identity among all thioesterases was observed in sequence alignments, the secondary structure elements are mostly well conserved and, therefore, it is expected that the active site architecture of those enzymes remains homologous. Investigations involving protein superfamilies have shown that the mode of catalysis, the active site location, as well as residues involved in substrate recognition and catalysis are indeed frequently conserved among these evolutionarily related proteins^35–37^. This explains the relative low sequence homology between *Sa*DHNA and other known thioesterases, even though the quaternary structures and, in particular the positions of the respective active sites and residues within the interface region forming the dimers, are homologous.

Thioesterases from *E. coli Ec*YbgC as well as *Haemophilus influenzae Hi*YbgC are more active for short acyl chain substrates, in contrast to *Helicobacter pylori Hp*YbgC, which is more active towards long acyl chains, e.g. palmitoyl- and stearoyl-CoA^38^. Although all of the enzymes possess the same HotDog fold class I, *Ec*YbgC, *Hi*YbgC, *Hp*YbgC as well as *Sa*DHNA have some differences in their structures, which explains a divergence in the substrate specificity. In fact, we observed the presence of a long tunnel associated with the binding site of the acyl moiety of the substrate for *Hp*YbgC, which is absent in the *Hi*YbgC. We detected a similar situation for *Sa*DHNA, which is more active towards long acyl chains (stearoyl-CoA) in comparison to a short chain (crotonyl-CoA). The structure of *Sa*DHNA reveals that activity towards long acyl chains is associated with the presence of an extended tunnel with a hydrophobic nature, involving residues Leu35, Ile38, Tyr45; Met48, Leu122, Tyr125 and Phe126 (Fig. S3), where the long acyl chain of a stearoyl-CoA may point towards this hydrophobic tunnel and is stabilized mostly through hydrophobic non-covalent interactions^31^.

Considering all known homologous thioesterase structures, investigations performed for the native *Ps*4HBT and a D17N *Ps*4HBT mutant revealed functional residues involved in the thioesterase activity of *Sa*DHNA. Residue His23 positioned in the N-terminus of the main α-HD helix (Ƞ2 and α1), and the substitution of Tyr24 in *Ps*4HBT, is likely to be responsible for the polarization of the thioester carbonyl carbon group by a hydrogen bond with the imidazole ring of the histidine sidechain. The carbonyl sidechain from the closest residue, Asp16 (positioned within the connecting β-turn loop between Ƞ1 and the main α-HD helix), may act as a nucleophile during the thioester bond cleavage. In fact, changing residue Asp16 (D16A) in *Sa*DHNA altered the rate of catalysis of the *Sa*DHNA thioesterase, resulting in a 300-fold decrease of the hydrolysis. This important result highlights the significance of this particular residue for the thioesterase activity of *Sa*DHNA. Nevertheless, the D16A substitution was not sufficient to completely inactivate *Sa*DHNA thioesterase activity, showing a divergent result compared to previously reported thioesterases^39–41^. This controversial result about the function of an aspartic acid in catalysis comparing *Ps*4HBT, Orf6 thioesterase and *Sa*DHNA indicates that the enzymatic mechanism of *Sa*DHNA may not occur according to a covalent catalysis, as observed for *Ps*4HBT, Orf6 thioesterase but more probably by catalysis involving a water molecule^30,42,43^. The mechanism involving the covalent catalysis is based on the proposed anhydride intermediate formation resulting from the nucleophilic attack performed by an acidic residue (aspartic or glutamic acid) in the active site, releasing the CoA thiol in the absence of a water molecule. On the other hand, the general base catalysis mechanism requires the directed activation of a water molecule which consequently acts as a nucleophile on the CoA thiol group^44,45^. Indeed, by a careful inspection of the native *Sa*DHNA structure, there is a water molecule close to BCA substrate and the sidechain of residue Asp16 located inside of the tunnel of the active site which could support the general base mechanism in *Sa*DHNA. Interestingly, although the substitution of the aspartic acid was sufficient to completely inactivate the thioesterases from *P. profundum* and *Pseudomonas* 4HBT, for *Sa*DHNA this mutant had substantially decreased activity but was not fully inactivated. This surprising result indicates that *Sa*DHNA might use the aspartic acid together with another residue as an alternative during thioesterase activity. Hydrolases, including thioesterases, frequently use the Ser-His-Asp catalytic triad to perform bond cleavage. Within this triad, aspartic acid is an important activator during nucleophile attack, followed by serine and histidine residues. The imidazole ring of histidine possesses a pK_a_ of approximately 6 to 7 which allows this residue to switch between protonated and unprotonated states at a physiological pH. This property enables histidine to participate in general acid–base catalysis and to enhance the nucleophilicity of the hydroxyl and thiol groups. Protonated nitrogen of the imidazole ring can act as a general acid while unprotonated nitrogen acts as nucleophile, and consequently, performs as a general base^46^. In the absence of aspartic acid at the active site, nitrogen from the imidazole ring of His23 might abstract a proton of the nucleophile (a water molecule of *Sa*DHNA native structure closed to the proximity of BCA substrate) and henceforward induce the nucleophilic attack on the carbonyl carbon of the polarized substrate (electrophile) as modelled in Fig. S4. On the other hand, altering residue Glu31 (E31N) resulted in no detectable activity. The orientation of the uncharged polar sidechain of an asparagine residue might interfere with the binding of substrate in the active site, in which Glu31 is more likely to act only as a supportive residue required for the substrate binding and not be involved in the thioesterase activity. Initial evidence obtained from our investigations support the general base catalysis.

Finally, peptide inhibitors were successfully screened by assays and docking studies using the atomic structure of *Sa*DHNA. In general, stable peptide-protein interactions involve hydrogen bonds, as well as complementary interactions, such as hydrophobic van der Waals interactions, leading to a high selectivity and binding affinity^47^. Although designed peptide inhibitors based on atomic structures have demonstrated effectiveness to inhibit bacterial protein synthesis^48^ and transcription^49^, so far, no attempt has been made to rationally design peptide inhibitors of a DHNA thioesterase. In this study we designed two peptide ligands. During docking analysis Pep-1 was predicted to bind inside the *Sa*DHNA active site, producing a stable interaction via hydrogen bonds, as well as noncovalent interactions and via aromatic ring stacking (π stacking), which may also contribute to the peptide stability inside the binding pocket. This stable interaction might prevent the substrate from binding by blocking the active site entrance for substrates. On the other hand, Pep-2 was predicted not to bind inside the active site, but on the surface of *Sa*DHNA. In contrast to traditional drug target sites, *e.g*. enzyme active sites, protein surface regions are usually more flat and mostly have less well-defined binding pockets for small molecules or peptides^50,51^. Thoden and co-workers^22^ observed that the coenzyme A ribose of both 4-hydroxybenzoyl-CoA substrates and the 4-hydroxyphenacyl-CoA inhibitor were positioned in a cleft located on the solvated surface of the dimer. This important observation suggests that the interaction of Pep-2 with the *Sa*DHNA surface might interfere with binding of the nucleotide moiety of the substrate and is reflected in the overall thioesterase activity^52^. Our spectroscopy assay data provided support the inhibitory properties of the rationally designed peptides Pep-1 and Pep-2, which might be considered as potential lead compounds for further investigations to provide more insights about potential *Sa*DHNA inhibition mechanisms.

## Conclusion

The high-resolution structure of a 4-hydroxybenzoyl-CoA thioesterase, a key enzyme involved in the menaquinone biosynthesis pathway of *S. aureus*, resembled a Hotdog fold class I structure for the monomer. The quaternary structure of the *Sa*DHNA homo-tetramer has four putative active sites, each located within the interface regions of two monomers, and with functionally important residues Asp16, His23 from one monomer and Glu31, Tyr45 from the adjacent monomer. Enzymatic assays and mutagenesis studies demonstrated a preference towards long chain substrates, as well as the importance of the acidic residues Asp16 and Glu31 in the active site and for substrate binding, respectively.

Targeting the menaquinone synthesis pathway of antibiotic resistant bacteria, such as *S. aureus*, can be considered as a new and innovative approach for future drug development investigations, due to the absence of the vitamin K biosynthesis in humans. Further, the peptide ligands designed for initial inhibition studies provide promising information for future inhibitor development.

## Material and methods

### Cloning, expression and purification of *Sa*DHNA

The *DHNA* gene was amplified by PCR from *S. aureus* cDNA using the primers sequence presented in Supplemental Table S2. The PCR product was cloned via restriction enzyme *Bsa*I (New England BioLabs) into *E. coli* expression vector pASK-IBA3 (IBA Lifescience) and the gene sequence was verified using automated sequencing (GATC Biotech AG, Germany). *E. coli* expression strain BL21 (DE3) was heat shock transformed with the resulting expression vector *Sa*DHNA-IBA3 and plated on LB agar supplemented with 100 mg·mL^−1^ ampicillin. For protein expression, the cells were grown in 1 L of Terrific broth media supplemented with 0.4% (v/v) glycerol (final concentration) and 100 mg∙mL^−1^ ampicillin at 37 °C until reaching an optical density of 0.6 applying a wavelength of 600 nm. Protein over-expression was induced with 200 ng mL^−1^ anhydrotetracycline (IBA Lifescience) at 37 °C for 6 h. Afterwards, for protein purification, the cells were harvested for 1 h at 4000 × g, 4 °C, and then resuspended in 100 mM Tris–HCl pH 8.0, 150 mM NaCl, 1 mM EDTA, 1 mM PMSF and sonicated twice for 8 min on ice. Soluble proteins were separated from the cell debris by centrifugation for 1 h, 18,000×*g* at 4 °C and the supernatant was applied onto a gravity column containing Step-Tactin resin (IBA Lifescience) in the cold room. Unbound proteins were removed from the column by utilizing a washing buffer (WB) containing 100 mM Tris–HCl pH 8.0, 150 mM NaCl, 1 mM EDTA. The protein was eluted using WB supplemented with 2.5 mM D-desthiobiotin (IBA Lifescience). Eluted protein was dialysed against 100 mM sodium phosphate buffer pH 6.0, 100 mM NaCl, applied to a pre-equilibrated HiLoad 16/600 Superdex 200 (Cytiva, former GE Healthcare) gel filtration column. After dialysis in 100 mM sodium phosphate buffer pH 6, 150 mM NaCl, *Sa*DHNA-CoA thioesterase was concentrated for crystallization experiments until 10 mg mL^−1^, using an extinction coefficient of 41,370 M^−1^ cm^−1^, provided by Protparam program (http://web.expasy.org/protparam/).

### Thioesterase activity assay

The thioesterase activity of *Sa*DHNA was measured according to a protocol of Rodríguez-Guilbe and co-workers^36^. In a ELISA microplate the formation of 2-nitro-5-thiobenzoate anion (TNB2-) by the reaction of thiolate anion (RS^−^) with Ellman′s reagent (DTNB^2−^) and one mixed disulphide (R-S-TNB^−^) catalysed by purified *Sa*DHNA wild type (WT), D16A and E31N was followed by monitoring the change in absorbance of thionitrobenzoic acid (TNB) at 412 nm (extinction coefficient of 13.600 M^−1^ cm^−1^) using a TECAN GENios plate reader (XFLUOR4 Version: V 4.40, MTX Lab Systems, Inc, USA). The enzymatic assays were performed in a total volume of 200 μl at room temperature by incubating 10 μM of enzyme in 50 mM HEPES-K^+^ buffer, pH 7.5 and 1 mM 5,5ʹ-dithiobis-(2-nitrobenzoic acid) (DTNB) for 1 h, followed by addition of 100 μM stearoyl-CoA (“long chain”) or 1 mM crotonyl-CoA (“short chain”). All enzymatic assays were carried out in triplicates, from individual protein production and purification batches. Peptide binding- and inhibition assays of *Sa*DHNA were performed according to the before mentioned thioesterase activity assay, applying a constant peptide concentration of 100 μM. Control reactions were performed with the same reagents, without adding the substrate in order to detect nonspecific conversion of DTNB by the enzyme. In addition, uncatalyzed reaction rates were monitored by the combination of same reagents without the enzyme.

### Site-directed mutagenesis

In order to obtain more information regarding the putative interactions identified for D16 and E31 and the substrate benzoyl-CoA obtained by superimposition using the homologous structure of *Ps*4HBT (pdb code 1LO9), as well as to obtain more insights about the structure–function relationship of *Sa*DHNA, site-directed mutagenesis for the residues D16 and E31 was performed by plasmid PCR amplification, according to Edelheit (2009)^53^. Corresponding PCR was performed by amplification of the parental plasmid DNA in two PCR tubes and adding the forward or the reverse primer, as listed in supplemental table S2, using the Q5 High fidelity DNA polymerase (New England Biolabs). After PCR, the reaction product was combined into one single tube, denaturated by heat to separate the recently synthesized DNA strain from the template and cooled down gradually to allow the annealing of the complementary chains. The original DNA template was digested by adding the restriction enzyme DnpI (Thermo Fisher Scientifc) and as a final step, used to transform BL21 (DE3) competent cells. Sanger sequencing (GATC Biotech AG, Germany) was performed to verify the sequence of the purified DNA plasmids.

### Crystallization, heavy atom derivative, data collection and structure determination

X-ray suitable crystals of *Sa*DHNA were obtained applying the sitting drop vapor-diffusion technique mixing in a ratio of 1:1 protein solution (10 mg·mL^−1^) and reservoir solution consisting of 100 mM HEPES-Na^+^ pH 7.0, 1.0 M lithium sulphate, equilibrated against 300 µL of reservoir solution utilizing MRC Maxi 48-wells plates (Molecular dimensions, UK) at 293 K. For cryo-cooling prior to X-ray data collection crystals were transferred to a new reservoir solution containing 15% (v/v) glycerol and flash-cooled in a nitrogen stream at 100 K. Diffraction data for native *Sa*DHNA were collected at the EMBL beamline P13 at PETRA III (DESY, Hamburg). In order to obtain phase information, heavy atom derivative soaking was performed using a final concentration of 1.25 mM potassium tetrachloroplatinate II (Hampton Research, USA), added to the crystal droplet 24 h before X-ray data collection. A platinum-SAD single-wavelength anomalous dispersion/diffraction (SAD) dataset was collected at 1.072 Å wavelength and up to 2.0 Å resolution at the EMBL beamline P14 at PETRA III (DESY, Hamburg) and was used for heavy atom localization and subsequent phasing. *Sa*DHNA and derivative data were indexed, integrated using the XDS software package^54^ and were scaled using the program AIMLESS from the *CCP*4i software suite^55–57^. In order to obtain phase information, the EMBL-HH Automated Crystal Structure Determination Platform Auto Rickshaw (EMBL Hamburg, Germany) (www.embl-hamburg.de/Auto-Rickshaw/)^58^ was used. Afterwards, successive rounds of model building and refinement were performed using the program *REFMAC5,* version 5.8.0131^59^ from *CCP*4i and the program *Coot* version 0.8.1^60^ for model building. All structure figures were generated using the PyMOL software suite version 1.3 and the final *Sa*DHNA structure was deposited at the Protein Data Bank (pdb code 6FDG).

### Small Angle X-ray Scattering (SAXS) measurements

Small-angle X-ray scattering data of monodispers *Sa*DHNA at concentrations from 0.8 to 2.5 mg ml^−1^ were collected at EMBL beamline P12 at the storage ring PETRA III (DESY, Hamburg, Germany). The monodispersity of the sample solutions was verified prior to SAXS data collection applying DLS using a SpectroLight 300 instrument (Xtal Concepts, Germany). Protein was applied in 100 mM sodium phosphate buffer pH 6.0 and 150 mM NaCl with a sample volume of 25 µl at 10 °C. SAXS data were collected at a sample-detector distance of 3.1 m, a wavelength of λ = 0.124 nm viand applying a 2D photon-counting Pilatus 2 M pixel detector (Dectris) with the momentum transfer ranging from 0.03 nm^−1^ < s < 5 nm^−1^ (s = 4π sinθ λ^−1^, where 2θ is the scattering angle). Data were normalized to the intensity of the transmitted beam and radially averaged. Scattering amplitudes from 20 successive X-ray exposures of 45 ms each were averaged and subtracted from the average of 40 buffer exposures. The Guinier region, radius of gyration R_g_ and the particle pair-distance distribution function p(r), which further provides the maximum dimension D_max_ of the protein, were obtained and evaluated applying the program PRIMUSQT, part of the ATSAS software suite^61^. Low-resolution chain-like ab initio shapes of *Sa*DHNA showing tetramer symmetry were subsequently generated using the composite scattering curves applying the program GASBOR^62^ and using a total number of 620 amino acid dummy spheres and 611 water molecules.

### Docking investigations, peptide rational design and synthesis

Docking studies were carried out with *Sa*DHNA homodimer using the BioLuminate software from the Schrödinger suite (Schrödinger, LLC, New York, 2021). The peptides were designed based on the structure of the natural substrate 1,4-dihydroxy-2-naphthoyl-CoA and used for peptide docking calculation applying the BioLuminate tool using the Molecular Mechanics/Generalized Born Surface Area (MM/GBSA) method to calculate the free binding energy^63^. Afterwards, peptides, Pep-1 and Pep-2, with the lowest scores obtained from the calculated free energy binging were synthesized and used for further in vitro inhibition assays.

### Investigation inhibitory activity of specific peptides *Sa* using fluorescence spectrophotometry

Fluorescence measurements were performed using a Cary eclipse fluorescence spectrophotometer coupled with a peltier temperature control (Agilent, USA). All assays were carried out in 1 mL cuvettes at 22 °C with excitation/emission slits at 20 nm each. The applied excitation wavelength was 290 nm and emission spectra were collected in the range of 300 up to 420 nm in increments of 1 nm. *Sa*DHNA quenching experiments were performed with two peptides (Pep-1: YGSDGR and Pep-2: EGEYE, using a concentration range from 60 up to 1000 nM. *Sa*DHNA at a concentration of 60 nM in 100 mM sodium phosphate buffer pH 6.0, 150 mM NaCl was applied and small volumes of the stock peptide solution were sequentially added to the cuvette for quenching analysis. Each *Sa*DHNA-peptide assay was performed five times. As a control the fluorescence of all buffer and all peptide solutions were measured to correct the observed fluorescence accordingly. The quenching process was assessed by the Stern–Volmer theory^64^.

## Supplementary Information

Supplementary Information.
