# Supplementary material for: Structure and activity of the DHNA Coenzyme-A Thioesterase from Staphylococcus aureus providing insights for innovative drug development
Source: Sci Rep. 2022 Mar 12;12:4313. doi: 10.1038/s41598-022-08281-2 (PMC8918352; doi:10.1038/s41598-022-08281-2)
Supplement: Supplementary file 1 — Supplementary Information. [file 41598_2022_8281_MOESM1_ESM.docx]

**Supplementary material**

**
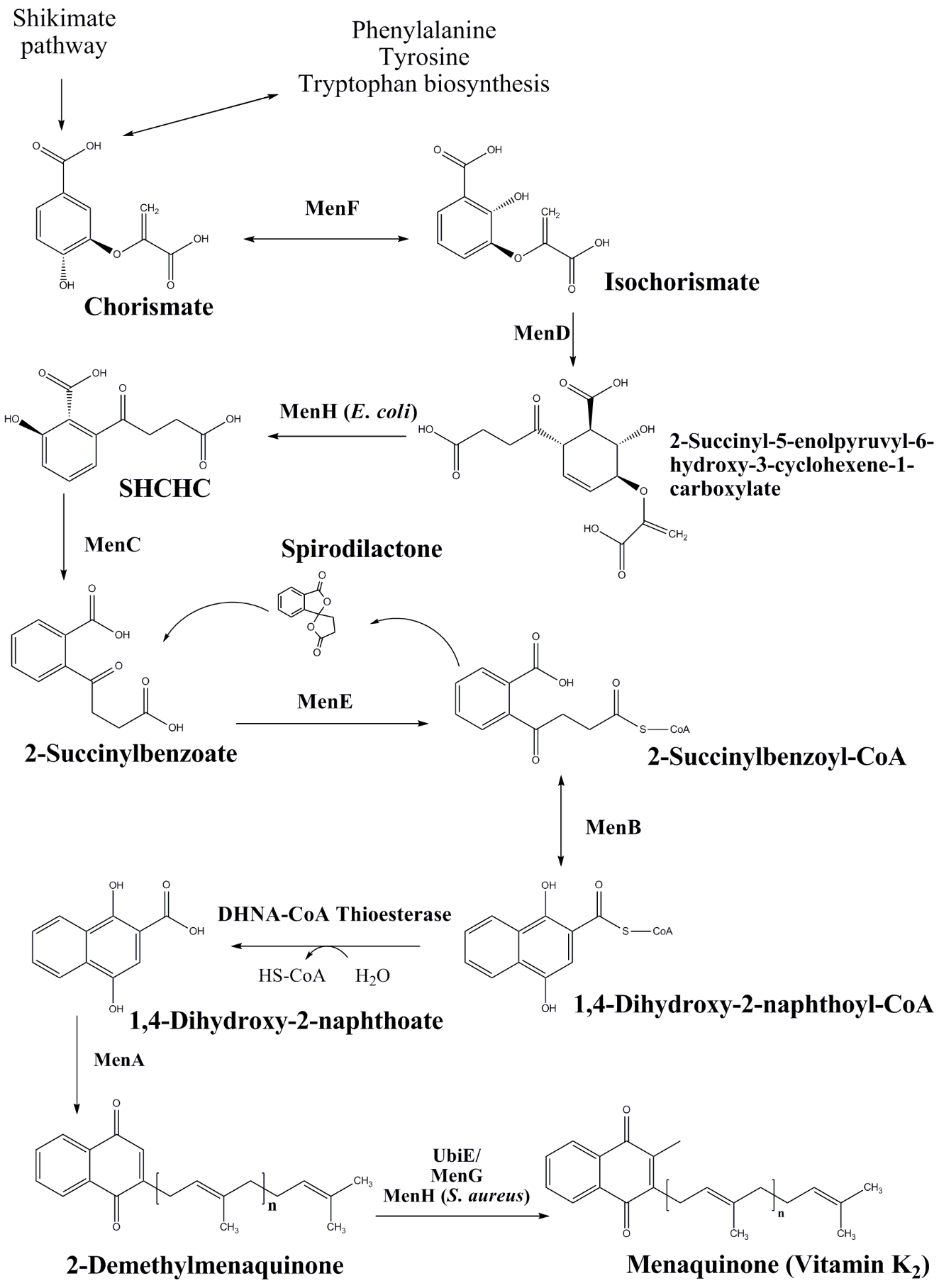
**

**Figure S1**. A classical overview of the menaquinone pathway. The figure was created using ChemDraw program (PerkinElmer Inc.) based on the Kyoto Encyclopedia of Genes and Genomes (KEGG)^65^.


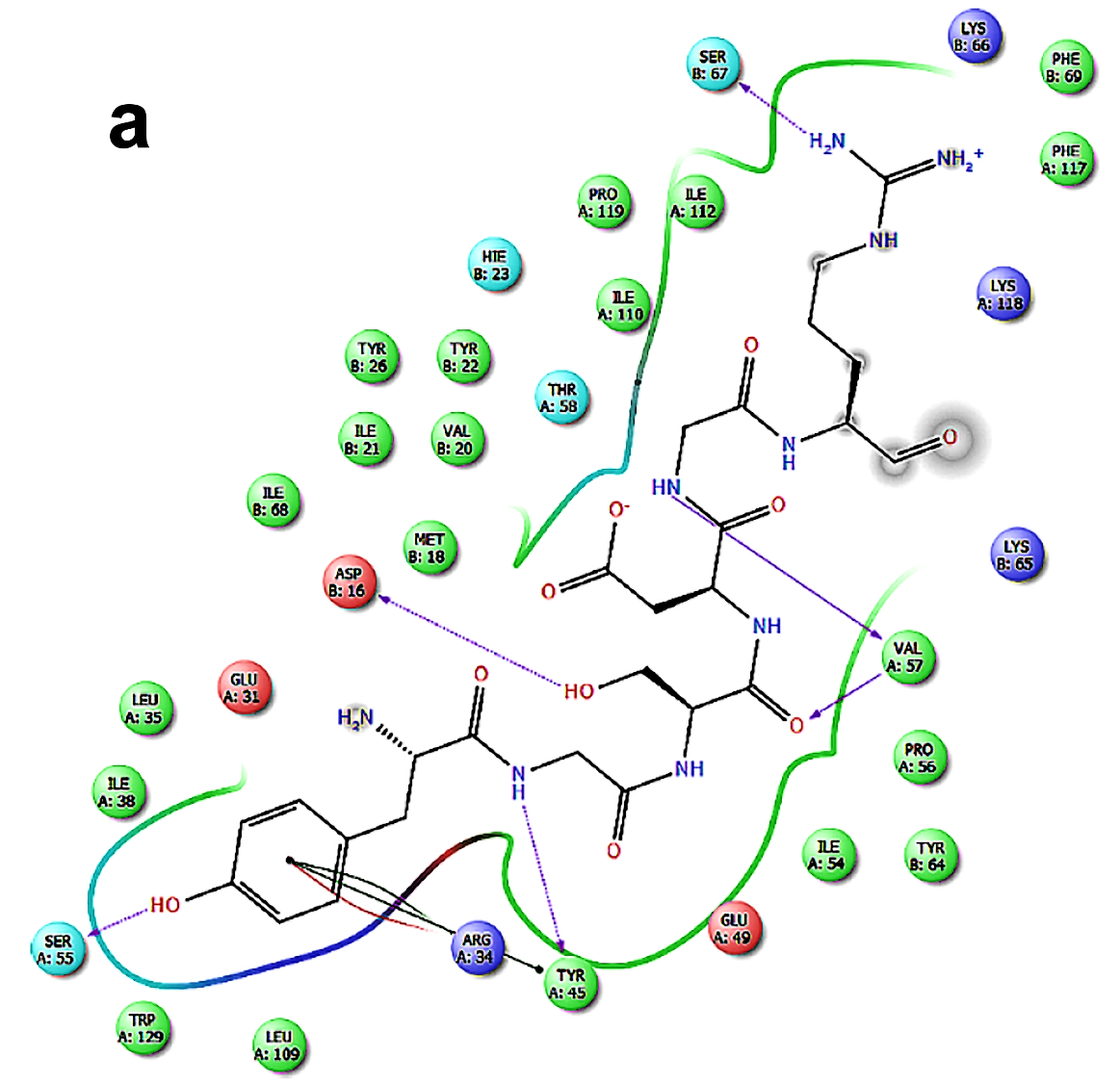

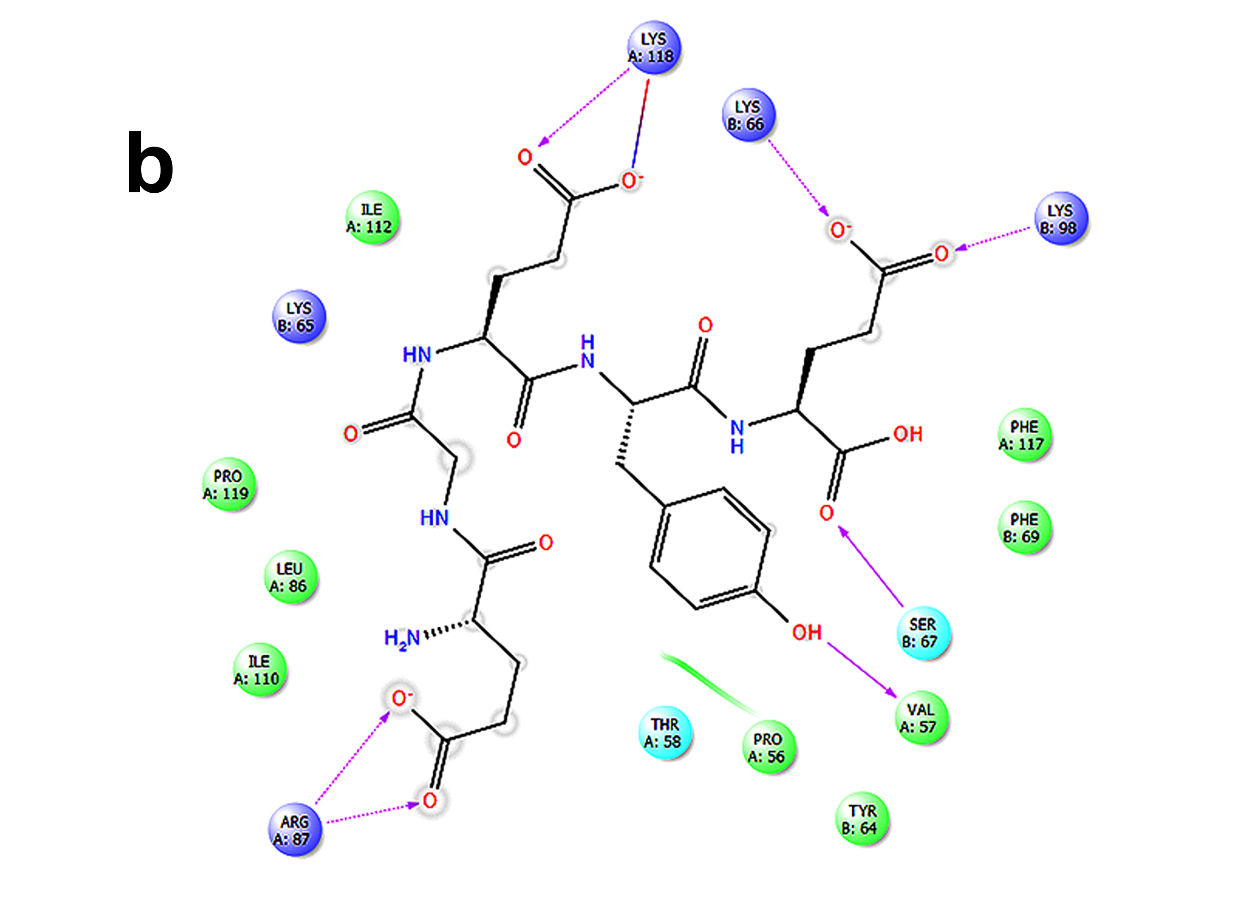


**Figure S2.** Ligand plots of Pep-1 (**a**) and Pep-2 (**b**) as predicted by *in silico* docking studies. Residues involved in interactions are shown and colored as negatively charged (pink), positively charged (purple), hydrophobic (green), and polar (cyan). Magenta arrows indicate hydrogen bonds and other arrows indicate π-stacking interactions.

**
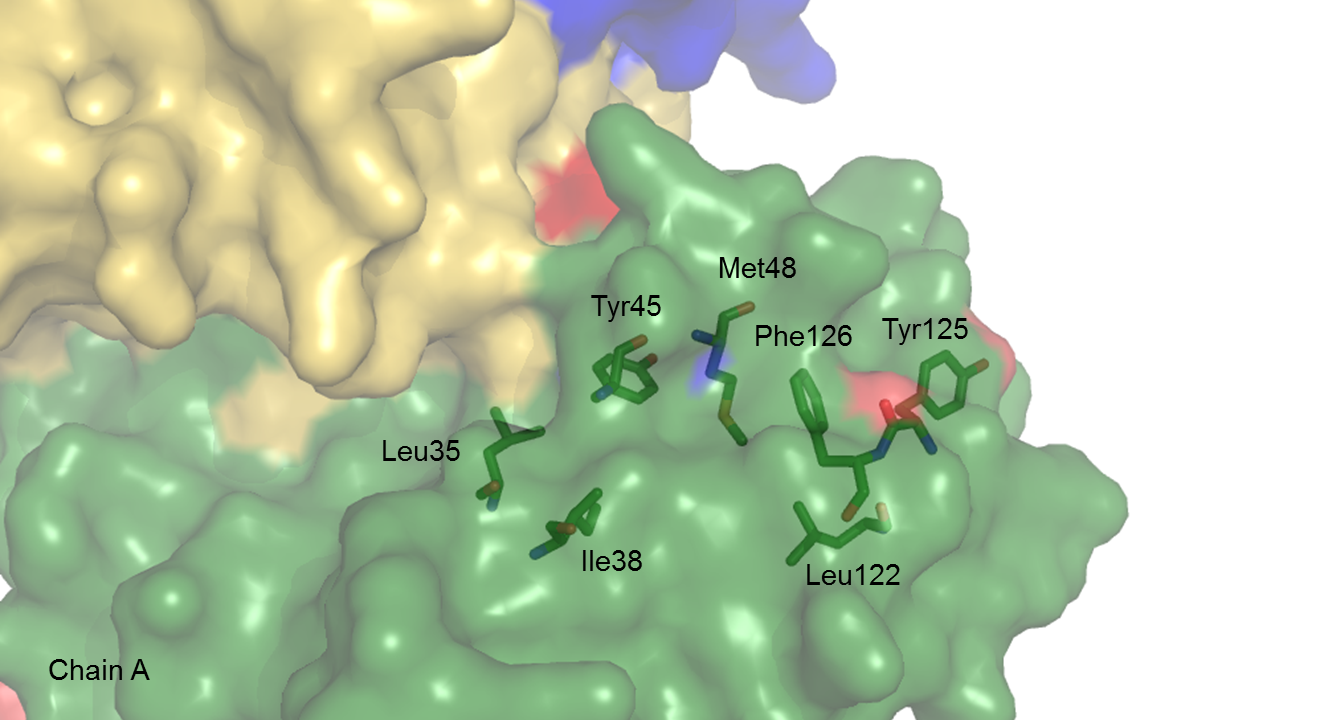
**

**Figure S3.** Elongated hydrophobic tunnel reaching the surface of *Sa*DHNA (chain A; green), which is supposed to influence the substrate specificity by determining the preferred length of the substrate acyl chain. Residues involved in substrate interactions are labeled. A homologous surface structure of functionally related enzymes was discussed by Pidugu *et al.*, 2009.

**
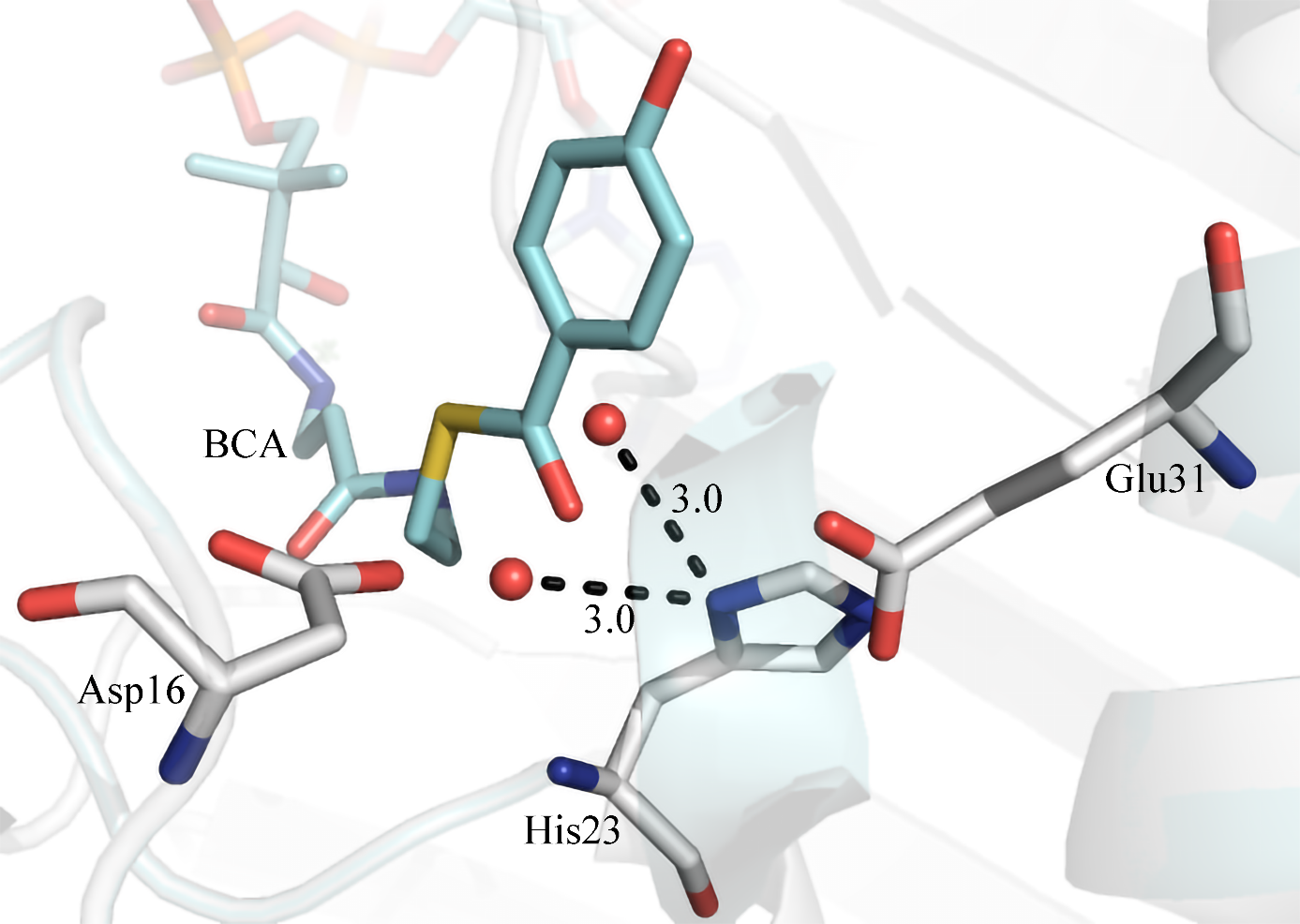
**

**Figure S4.** Model for proposed substrate interaction with *Sa*DHNA (light grey) with the modelled substrate (4-hydroxybenzoyl CoA - BCA), molecule in the center. Nitrogen from the imidazole ring of His23 may act as a general base, catalyzing by abstracting a proton of the nucleophile (water molecule) and inducing the nucleophilic attack upon the carbonyl carbon of a polarized substrate (light cyan). Water molecules shown are originated from the native *Sa*DHNA structure. Distances are provided in Å.

**Table S1.** Enzymatic activity of *Sa*DHNA WT and its mutants D16A and E31N. Values are in nmol TNB/min/mg protein and converted using the Beer equation and using the extinction coefficient at 412 nm for thionitrobenzoic acid (TNB), 13.6 mM^-1^ cm^-1^. (ND: not detectable).

| **Substrate** | ***Sa*DHNA WT** | **D16A** | **E31N** |
| --- | --- | --- | --- |
| Stearoyl-CoA (C_18:0_) | 128 ± 0.054 | 0.43 ± 0.02 | ND |
| Crotonyl-CoA (C_4:1_) | 0.27 ± 0.002 | - | - |

**Table S2.** Primer sequences used for cloning and site-directed mutagenesis in 5’ →3’. Mutation sites are in bold for mutagenesis primers.

| *Sa*DHNA IBA3-Forward | 5’-GCGCGCGGTCTCGAATGATATATAGTATTACAGAAATAG-3’ |
| --- | --- |
| *Sa*DHNA IBA3-Reverse | 5’-GCGCGCGGTCTCAGCGCTTAAAGAATCAATACCATCCATTATC-3’ |
| *Sa*DHNA-D16A- Forward | 5’-GCGCGTTATGCTGAAACT**GCT**AAGATGGGTGTAATTTATC-3’ |
| *Sa*DHNA-D16A- Reverse | 5’-GATAAATTACACCCATCTT**AGC**AGTTTCAGCATAACGCGC-3’ |
| *Sa*DHNA-E31N- Forward | 5’-GCAACTTGGTTT**AAC**GTTGCGCGGTTGG-3’ |
| *Sa*DHNA-E31N- Reverse | 5’-CCAACCGCGCAA**CGT**TAAACCAAGTTGC-3’ |
